# Correlation between single nucleotide polymorphisms in TLR4, LTA and RFP175 genes and susceptibility to invasive bacterial infections in infants under three months of age: A case control study

**DOI:** 10.12669/pjms.41.12.12651

**Published:** 2025-12

**Authors:** Ali Yurtseven, Enise Avci Durmusalioglu, Benay Turan, Eylem Ulas Saz

**Affiliations:** 1 Ali Yurtseven, MD, Associate Professor, Department of Pediatrics, Division of Emergency Medicine, Faculty of Medicine, Ege University, Izmir, Turkiye; 2 Enise Avci Durmusalioglu, MD, Department of Pediatrics, Division of Genetics, Faculty of Medicine, Ege University, Izmir, Turkiye; 3 Benay Turan, MD, Department of Pediatrics, Faculty of Medicine, Ege University, Izmir, Turkiye; 4 Eylem Ulas Saz, MD, Professor, Department of Pediatrics, Division of Emergency Medicine, Faculty of Medicine, Ege University, Izmir, Turkiye

**Keywords:** TLR4 gene, LTA gene, RFP175 gene, Infant, Invasive bacterial infections, Single nucleotide polymorphism

## Abstract

**Objective:**

This study investigates the correlation of TLR4 rs2149356, LTA rs2229094 and RFP175 rs1585110 genes single nucleotide polymorphisms (SNPs) with invasive bacterial infections (IBIs) in neonates and young infants.

**Methodology:**

We conducted a prospective, case-control study at Ege University Faculty of Medicine, Izmir, Turkiye, between January 2021 to December 2023. Infants under 90 days of age admitted with confirmed IBIs (cases) or non-infectious conditions (controls) were included. Genomic DNA was extracted from peripheral blood samples and next-generation sequencing was used to analyze selected SNPs in TLR4, LTA and RFP175 genes. Clinical and demographic characteristics, as well as SNP frequencies, were compared between groups.

**Results:**

A total of 200 infants were enrolled (100 cases, 100 controls). Most infections were urinary tract infections (n=84), 84%), with smaller numbers of gastroenteritis (n=6, 6%), bacteremia (n=5, 5%), pneumonia (n=3, 3%), and meningitis (n=2, 2%). No significant differences were found in clinical characteristics or SNP frequencies between groups.

**Conclusions:**

This study found no association between the investigated SNPs in TLR4, LTA and RFP175 and susceptibility to IBIs in infants under three months. Larger studies are needed to clarify the genetic contribution to early-life infections.

***Clinical Trial Registration:*** NCT06985160

## INTRODUCTION

Despite medical advances, invasive bacterial infections (IBIs) remain a major cause of mortality in neonates and young children, accounting for nearly half of all deaths in children under five worldwide.^1^ The first three months of life represent a particularly vulnerable period, during which 10-20% of febrile infants are diagnosed with an IBI, a rate significantly higher than that observed in older age groups.^2^,^3^ Although, multiple risk factors have been proposed, the underlying mechanisms behind why some infants develop IBIs while others do not remain unclear. Increasing evidence suggests a role for genetic susceptibility.^4^

Genetic polymorphisms have been linked to increased vulnerability to IBIs in several studies.^5^-^7^ Toll-like receptors (TLRs), particularly expressed in monocytes, macrophages and dendritic cells, are essential components of the innate immune system. By recognizing pathogen-associated molecular patterns, TLRs activate intracellular signaling cascades that result in inflammatory responses vital for pathogen clearance.^8^-^10^

TLRs play a central role in microbial recognition and immune activation.^11^ Lymphotoxin-alpha (LTA), formerly known as TNF-beta, is another important immune modulator. Polymorphisms in the LTA gene have been inversely associated with sepsis susceptibility.^12^ Similarly, RING finger proteins (RFPs), which function as ubiquitin ligases, have been implicated in the pathogenesis of bacterial infections through genetic variation.^13^

Some studies report that the TLR4 rs2149356 G/T and LTA rs2229094 C genotypes are associated with reduced IBI risk, while the RFP175 rs1585110 C/T genotype is linked to increased susceptibility.^13^,^14^ However, most such studies focus on adults or include broad pediatric age groups.^9^-^13^ Since IBIs are most frequent during the first three months of life, research specific to this age group is critical yet limited.^2^

This study aimed to examine the association between TLR4 rs2149356, LTA rs2229094 and RFP175 rs1585110 polymorphisms and the risk of invasive bacterial infections in infants under three months of age.

## METHODOLOGY

This prospective, case-control study was conducted at Ege University Faculty of Medicine, a tertiary academic hospital in Izmir, Turkiye, between January 2021 to December 2023. The study included one hundred infants aged 0-3 months diagnosed with invasive bacterial infections (IBIs) and one hundred controls admitted with non-infectious conditions such as trauma, infantile colic, or hyperbilirubinemia. Infants in the control group with any signs of infection were excluded.

### Ethical Approval:

***The*** study was approved by the Scientific Research Ethics Committee of Ege University Faculty of Medicine (Date July 8, 2020 / No. 20-7T/88) and written informed consent was collected from parents or legal guardians before enrollment, in accordance with the Declaration of Helsinki.

IBIs in this study comprised meningitis, pneumonia, sepsis, bacteremia, urinary tract infections (UTIs) and bacterial gastroenteritis. Diagnostic criteria were as follows: meningitis presence of bacteria identified by culture or PCR in cerebrospinal fluid (CSF), or CSF findings consistent with bacterial infection (elevated leukocytes with neutrophil predominance, low glucose, and high protein); pneumonia compatible clinical presentation (fever, tachypnea, respiratory distress, or auscultatory findings) in combination with radiographic evidence of pulmonary infiltrates or consolidation; bacteremia-positive blood culture; UTI- growth of a single bacterial species ≥10^3^ CFU/mL from a sterilely obtained catheterized urine specimen, accompanied by compatible clinical signs; and gastroenteritis isolation of enteric bacterial pathogens from stool cultures in infants presenting with diarrhea and systemic symptoms.

No additional blood was drawn for the study. Instead, leftover blood samples initially collected for routine diagnostic purposes in the emergency department were retrieved from the laboratory after clinical tests were completed and repurposed for genetic analysis.

### Genetic analysis:

Genomic DNA was extracted from whole blood samples using the QIAamp DNA Mini Kit (Qiagen) following the manufacturer’s protocol. DNA purity and concentration were assessed with a NanoDrop ND-1000 Spectrophotometer and only high-quality samples were used for sequencing. Primers targeting the polymorphisms TLR4 rs2149356 (c.261-468T>G), LTA rs2229094 (c.37T>C) and RFP175 rs1585110 (c.246+8853G>A) were designed using Primer3 software. Primer design was optimized for specificity, GC content and melting temperature to amplify regions of approximately 300-500 base pairs.

The target regions were amplified via polymerase chain reaction (PCR). Each 25 µL reaction included genomic DNA, specific primers, MgCl_2_, Taq polymerase and dNTPs. PCR conditions were optimized to ensure high-fidelity amplification. Amplified products were verified through 2% agarose gel electrophoresis and visualized under UV light. Following successful amplification, PCR products were sequenced using the Illumina MiSeq platform. The sequencing yielded FastQ files, which were converted into BAM format for alignment and variant analysis. The Integrative Genomics Viewer (IGV) was employed for visualization and identification of allelic variants. Polymorphisms of interest-TLR4 rs2149356, LTA rs2229094 and RFP175 rs1585110-were annotated for each individual in the study. Sequence analysis focused exclusively on these loci, as per the study objectives.

### Data analysis:

Allelic and genotypic frequencies of TLR4 rs2149356, LTA rs2229094 and RFP175 rs1585110 were compared between IBI cases and controls. Genotypic distributions were evaluated under dominant and recessive models: the dominant model compared carriers of at least one variant allele to homozygous wild-type, while the recessive model compared homozygous variants to the combined non-variant genotypes. Hardy-Weinberg equilibrium (HWE) was assessed using Fisher’s exact test by comparing observed and expected genotype frequencies. Power analysis indicated that 188 participants per group would be required to detect significant differences (power: 80%, alpha: 0.05).^4^,^5^,^7^,^14^

### Statistical analysis:

It was performed using IBM SPSS Statistics version 25. Categorical variables were analyzed using the chi-square test and associations were expressed as odds ratios (ORs) with 95% confidence intervals. A p-value < 0.05 was considered statistically significant.

## RESULTS

The study included 200 infants aged 0-3 months, with one hundred diagnosed with invasive bacterial infections and one hundred age-matched controls with non-infectious conditions. The mean age was 58.5 ± 29 days and 52.5% (n=105) were male. Prematurity was observed in 11.5% (n=23) and 42.5% (n=85) had received one dose of *pneumococcal* and *Haemophilus influenzae Type-B* vaccines. Baseline characteristics did not significantly differ between the groups in terms of age (p=0.951), sex (p=0.257), prematurity status (p=0.658), or vaccination status (p=0.775) (Table-I).

**Table-I:** The characteristics of the patient and control groups.

Cases’ Characteristics	Patient group N=100	Control group N=100	OR (95% CI)	P-value
Age, days (mean, ± SD)	58 ± 24	59 ± 23	-	0.951
***Gender, (n, %)*** Female Male	52(52) 48(48)	43(43) 57(57)	1.43 (0.82-2.50)	0.257
Prematurity (+) - no.(%)	13 (13)	10 (10)	1.30 (0.60-2.82)	0.658
Single dose vaccine (+) - no.(%)	41 (41)	44 (44)	1.13 (0.65-1.98)	0.775

CI = Confidence interval; OR = Odds ratio.

Among infected infants, urinary tract infections (UTIs) were most common (84%), followed by acute gastroenteritis (6%), bacteremia (5%), pneumonia (3%) and meningitis (2%). *Escherichia* coli was the leading uropathogen (60 cases), with *Klebsiella pneumoniae* (13), *Enterococcus faecalis* (8) and *Proteus mirabilis* (3) also identified. Gastroenteritis pathogens included *Campylobacter jejuni* (n=4) and *Salmonella enteritidis* (n=2). Bacteremia cases involved *Group-B Streptococcus* (n=2), E. coli *Escherichia coli* (n=2) and *Neisseria meningitidis* (n=1). Meningitis cases were confirmed with *Group-B Streptococcus* and *Neisseria meningitidis*.

Table-II presents genotype frequencies. No significant association was found between TLR4 rs2149356, LTA rs2229094, or RFP175 rs1585110 polymorphisms and susceptibility to invasive bacterial infections under either dominant or recessive genetic models.

**Table-II:** Genotype frequencies with significant differences in the selected gene polymorphisms between controls and children with invasive bacterial infections.

Gene and polymorphic alleles	IBIs group N=100 N(%)	Control group N=100 N(%)	HWE, χ^2^ IBIs P-value	HWE, χ^2^ Controls P-value	Outcome OR (95% CI)	P-value
***TLR4 rs2149356*** GG GT TT	42(42) 52(52) 6(6)	45(45) 41(41) 14(14)	0.051	0.353	1 (reference) 1.36 (0.75-2.44) 1.14 (0.96-1.37)	0.37 0.21
***Allelic distribution of TLR4 rs2149356***						
G T	136(68) 64(32)	131(66) 69(34)			1.12 (0.89-1.42)	0.74
***LTA rs2229094*** TT CT CC	55(55) 37(37) 8(8)	51(51) 44(44) 5(5)	0.615	0.245	1 (reference) 1.42 (0.49-4.09) 1.74 (0.61-4.93)	0.56 0.37
***Allelic distribution of LTA rs2229094***						
T C	147(74) 53(26)	146(73) 54(27)			1.03 (0.95-1.12)	1.00
***RFP175 rs1585110*** CC CT TT	83(83) 13(13) 4(4)	88(88) 7(7) 5(5)	0.002	< 0.001	1 (reference) 1.05 (0.78-1.32) 1.31 (0.76-2.26)	0.77 0.42
***Allelic distribution of RFP175 rs1585110***						
C T	179(90) 21(10)	185(92) 15(8)			1.45 (0.71-2.81)	0.38
Coexistence of the SNPs	8(8)	6(6)			1.37 (0.46-4.12)	0.59

CI = Confidence interval; HWE= Hardy-Weinberg equilibrium; IBI=Invasive bacterial infection; OR = Odds ratio; SNP= single nucleotide polymorphism

## DISCUSSION

This study is the first, to our knowledge, to investigate the association between TLR4 rs2149356, LTA rs2229094 and RFP175 rs1585110 gene polymorphisms and susceptibility to invasive bacterial infections (IBIs) exclusively in infants aged 0-3 months-a population with the highest incidence and mortality related to IBIs. Our results showed no significant associations between these SNPs and IBI susceptibility across dominant and recessive genetic models.

While previous studies, such as those by Esposito et al., suggested potential associations between these polymorphisms and IBI risk in a broader pediatric age range, our findings suggest that such genetic associations may not hold during the neonatal and early infancy period.^14^-^16^ Notably, Esposito et al. reported a borderline increased IBI risk with the C/T genotype of RFP175 rs1585110 and a protective effect for the G/T genotype of TLR4 rs2149356 and the C allele of LTA rs2229094. However, their findings may reflect age-related differences in immune system maturity or pathogen exposure, highlighting the importance of age-specific genetic studies in infectious disease susceptibility.

Our findings are also in line with larger studies, such as Abu-Maziad et al., which examined numerous immune-related SNPs and found no consistent association with neonatal sepsis risk.^17^ Moreover, a meta-analysis of over 6,500 patients similarly found no significant link between TLR4 gene polymorphisms and sepsis susceptibility.^18^ Consistent with prior reports, urinary tract infections were the most common IBI in our study.^2^,^19^ The reduced incidence of other IBIs such as bacteremia and meningitis may reflect the impact of pneumococcal and Hib vaccination programs.

### Strength of the study:

The study’s major strength lies in its focused cohort-infants in the first 90 days of life-alongside the use of stringent infection definitions, next-generation sequencing for accurate SNP detection and the inclusion of a well-matched control group. Furthermore, sample reuse from routine clinical testing minimized invasiveness, adhering to ethical standards for pediatric research. The study also contributes to the limited pool of genetic data specifically addressing innate immune polymorphisms in the neonatal period.

### Limitations:

It was conducted at a single center, which may limit generalizability. Patients were evaluated by different clinicians, possibly introducing variability in severity classification. Only hospitalized patients were included, excluding milder cases managed at home. Although the total sample was sizable, the majority had UTIs, while fewer had bacteremia or meningitis, our ability to detect genotype–phenotype associations specific to these infection types was limited. Genetic susceptibility may differ by pathogen and infection site; therefore, future studies with more balanced infection distributions are warranted.

## CONCLUSION

Our research contributes to the body of evidence suggesting that the polymorphisms TLR4 rs2149356, LTA rs2229094 and RFP175 rs1585110 do not influence the development of IBIs in infants under three months. Future research should focus on larger, multicenter cohorts to validate these findings, explore other immune-related gene variants and investigate gene-expression patterns or epigenetic factors. Integrating genomic data with clinical and microbiological profiling may help refine risk prediction models for IBIs in early infancy.

### Recommendations:

Future studies should focus on multicenter, population-based cohorts to confirm these findings in more diverse clinical and genetic backgrounds. Integration of genomic data with transcriptomic and proteomic profiles may also help elucidate age-dependent mechanisms of immune activation. Ultimately, large-scale, age-stratified analyses are needed to determine whether early-life infection susceptibility is driven more by host genetic variation, epigenetic regulation, or environmental exposures.

### Authors’ Contributions:

**AY:** led the study and prepared the manuscript.

**AY, BT and EUS:** designed the study and did literature search.

**AY, EAD and BT:** took care of patients and collected data.

**AY and EAD:** Analyzed data.

**EUS:** did the critical revision of the manuscript.

**AY:** accepts full responsibility for the accuracy and integrity of the data and the final manuscript.

All authors have read and approved the final manuscript.
